# One-shot Cluster-Based Approach for the Detection of COVID–19 from Chest X–ray Images

**DOI:** 10.1007/s12559-020-09774-w

**Published:** 2021-03-02

**Authors:** V. N. Manjunath Aradhya, Mufti Mahmud, D. S. Guru, Basant Agarwal, M. Shamim Kaiser

**Affiliations:** 1 Department of Computer Applications, JSS Science & Technology University, Mysuru, 570006 India; 2 Department of Computer Science, Nottingham Trent University, Clifton, Nottingham NG11 8NS UK; 3 Medical Technology Innovation Facility, Nottingham Trent University, Clifton, Nottingham NG11 8NS UK; 4 Department of Studies in Computer Science, University of Mysore, Mysuru, 570006 India; 5 Department of Computer Science and Engineering, IIIT Kota, Rajasthan, India; 6 Institute of Information Technology, Jahangirnagar University, Savar, Dhaka 1342 Bangladesh

**Keywords:** Machine learning, Classification, Neural networks, GRNN, PNN, COVID-19, Chest X-rays

## Abstract

Coronavirus disease (COVID-19) has infected over more than 28.3 million people around the globe and killed 913K people worldwide as on 11 September 2020. With this pandemic, to combat the spreading of COVID-19, effective testing methodologies and immediate medical treatments are much required. Chest X-rays are the widely available modalities for immediate diagnosis of COVID-19. Hence, automation of detection of COVID-19 from chest X-ray images using machine learning approaches is of greater demand. A model for detecting COVID-19 from chest X-ray images is proposed in this paper. A novel concept of cluster-based one-shot learning is introduced in this work. The introduced concept has an advantage of learning from a few samples against learning from many samples in case of deep leaning architectures. The proposed model is a multi-class classification model as it classifies images of four classes, viz., pneumonia bacterial, pneumonia virus, normal, and COVID-19. The proposed model is based on ensemble of Generalized Regression Neural Network (GRNN) and Probabilistic Neural Network (PNN) classifiers at decision level. The effectiveness of the proposed model has been demonstrated through extensive experimentation on a publicly available dataset consisting of 306 images. The proposed cluster-based one-shot learning has been found to be more effective on GRNN and PNN ensembled model to distinguish COVID-19 images from that of the other three classes. It has also been experimentally observed that the model has a superior performance over contemporary deep learning architectures. The concept of one-shot cluster-based learning is being first of its kind in literature, expected to open up several new dimensions in the field of machine learning which require further researching for various applications.

## Introduction

The world has witnessed over 28.3 million cases and 913K deaths due to Corona Virus disease (COVID-19) outbreak as on 11 September 2020 [1]. With this pandemic, to combat the spreading of COVID-19, effective testing methodologies and immediate medical treatments are much required. Observations of clinical symptoms and analysis of radiography images are the diagnosis tools of COVID-19 detection. One of the major challenges with typical x-ray images is distinguishing COVID-19-infected images from that of various viral pneumonia versus infected images since they have similar appearance. It is quite difficult for radiologists to distinguish COVID-19 from other viral pneumonia and hence it can sometimes result with a wrong diagnosis, leading to a non-COVID viral pneumonia being falsely labeled as highly suspicious of having COVID-19 and vice versa.

With this backdrop, development of machine learning (ML)–based models to overcome these issues in classifying COVID-19 images has become an urgent requirement. Study on classifying and detecting the presence of pneumonia based on a ConvNet model is reported in [2, 3]. For early diagnosis of pneumonia, chest X-ray image models based on Xception and VGG16 are proposed in [4]. Experiments showed that VGG16 network outperformed Xception network. ConvNet models followed by different classifiers for the detection of normal and abnormal pneumonia X-ray images are recommended in [5]. COVIDX-Net model comprising of seven convolutional neural networks (CNNs) to diagnose COVID-19 is proposed in [6]. In [7], a three class problem considering Normal, COVID-19 viral, and non-COVID-19 viral cases is reported. A detection model based on generative adversarial network (GAN) and Deep Transfer Learning is proposed in [8]. Three deep learning models, such as AlexNet, GoogleNet, and ResNet18, were selected for investigation.

From the review of literature, we understand that in these days, researchers are employing machine learning (ML) in a variety of tasks including biological data mining [9, 10], image analysis [11, 27], financial forecasting [12], anomaly detection [13–15], disease detection [16, 17], natural language processing [18, 19], and assay detection [20]. More recent studies on COVID-19 detection using deep learning (DL) models can be seen in the literature [21]. These algorithms for detection/classification require huge amount of data. It is sometimes difficult to get more data due to limitations in medical field. Hence, it is more convenient to learn from a few examples and in this connection, one shot learning is to mimic the way human beings learn to make classification or prediction possible on a wide range of similar but new problems. The core constraint of this type of task is that the algorithm should decide on the class of a test instance after seeing just one example. In this work, we propose a model for detecting COVID-19 chest X-ray images, by introducing a new concept of one-shot cluster-based learning, which has greater advantages of learning from a few samples over any other existing deep learning–based models which learn from a huge number of samples. The following are the contributions of this work:

- Introduction of the notion of one-shot learning for efficient detection of COVID-19 chest X-ray images.
- Proposal of ensembling of GRNN and PNN classifiers using AND operation, which works on one-shot cluster-based learning to cope with the present pandemic situation.
- Exhaustive experimentation and comparative analysis of the results obtained against that of deep learning models.

## Proposed Pipeline

### One-shot Learning and Feature Representation

**Fig. 1:**
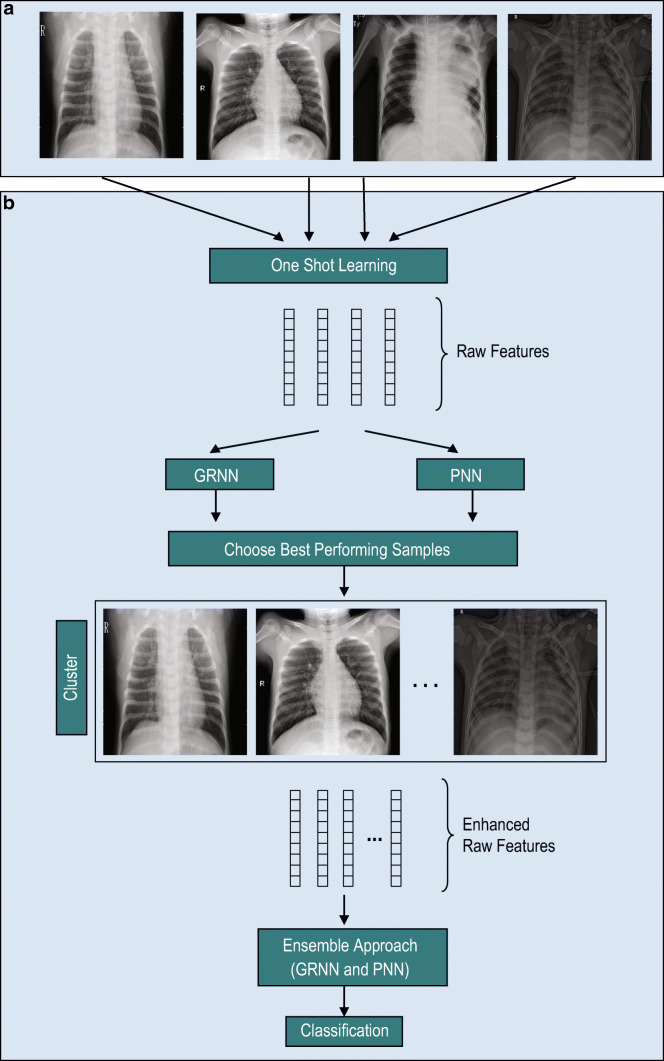
Block diagram of the proposed one-shot-based COVID-19 detection pipeline

The need for one-shot learning is guaranteed by following key considerations: (a). *Human Learning with few examples*: Learning in human happens with few examples. Human can relate new concepts to already learned concepts thereby enriching their domain knowledge. A model mimicking this is of great demand. (b). *Scalability constraints with deep learning*: Deep Learning has definitely set new benchmarks in performance for specific learning tasks like object recognition, language understanding, and machine translation. However, these algorithms need millions of training examples to build their intelligent behavior. (c). *Domains with sparse data*: In certain domains, availability of millions of data points for training the model is a challenge. In such applications, getting a large number of training examples have practical constraints and recognition needs to be performed with only a few available data points. Generally, in N*K shot learning, where *N* and *K* are respectively the number of classes and the number of samples in each class, we use *K* value reasonably big and very huge in deep learning–based approach, while in our one-shot learning, *K* is restricted to one sample. Figure 1 shows the block diagram of the proposed model. In this work, we introduced the notion of finding out a best performing samples in each class in terms their ability in distinguishing the samples belonging to different classes. We suggest to study the ability of a sample of a class in differentiating the samples of other classes by considering them one by one and feeding into classifiers. We adopt Generalized Regression Neural Network (GRNN) and Probabilistic Neural Network (PNN) for this purpose. Once the samples of each class are ranked based on their discrimination abilities, we recommend forming a cluster of randomly chosen number of top performing samples from each class, which shall later be used for one-shot learning of an ensembled classifier of GRNN and PNN. Here we adopt ensembling of GRNN and PNN at decision level using AND operation specifically as the designed system is expected behave like taking up a second opinion by a medical practitioner suiting the reality.

### Neural Network-Based Classifiers

Artificial Neural Networks (ANNs) are considered to be one of the important aspects of AI with a wide range of applications. ANNs are useful since they can learn from data and they have global approximation abilities. In recent past, Generalized Regression Neural Networks (GRNN) and Probabilistic Neural Network (PNN) have shown wide range of applications to solve real world problems. On the other hand, there is a consensus in machine learning community that support vector machine (SVM) is a most promising classifier due to its excellent generalization performance. However, SVMs for mulitclass classification problems are relatively slow and their training on a large data set is still a bottle-neck. The inherent advantage of GRNN/PNN is better in generalization and convergence properties when compared to that of other classifiers.

As the weights of these networks can be calculated analytically, GRNN are variants of the radial basis functions (RBF) network. GRNN uses a Gaussian activation function in the hidden layer and it is a single pass network, which consists of input, hidden, summation, and output layers. The number of input units depends on the total number of observation parameters, i.e., an input vector *I*. The input layer connected to the pattern layer consists of neurons provides training patterns and it outputs to the summation layer to perform normalization of the resultant output set. Each of the pattern layers is connected to the summation neurons and calculates the weight vector using the (1).

1 $$ \begin{aligned} F(I) &= \frac{{\sum}_{i=1}^{n} T_{i} W_{i}}{{\sum}_{i=1}^{n} W_{i}}, \quad \text{with}\\ W_{i} &= e^{[\frac{||I-I_{t} ||^{2}}{2h^{2}}]}, \quad \textrm{and \textit{T }is the target value}. \end{aligned}  $$

On the other hand, PNN is also a four-layer neural system, influenced by the Bayesian network. This approach has been studied well from the decades (1960s). As it models the Bayesian classifier misclassification rate is minimized. Bayes’ classifier is usually criticized due to lack of information about the class probability distributions and makes use of nonparametric techniques, whereas the inherent advantage of PNN has the better generalization and convergence properties when compared to that of Bayesian classifier in classification problems [22]. PNN is similar to that of supervised learning architecture, but PNN does not carry weights in its hidden layer. Each node of hidden layer acts as weights of an example vector. The hidden node activation is defined as the product of example vector *E* and input feature vector *F* given as *h*_*i*_ = *E*_*i*_ × *F*. The class output activations are carried out using the (2):

2 $$ S_{j}=\frac{{\sum}_{i=1}^{n}e^{\frac{h_{i}-1}{\phi^{2}}}}{N}  $$

where *N* is the number of example vectors belonging to class *S*_*j*_, *h*_*i*_ is hidden node activation, and *ϕ* is smoothing factor. PNN have some advantages compared to other neural architectures. PNN networks are usually faster to train, often more accurate and relatively insensitive to outliers. As PNN approaches Bayes classification it predicts accurate target probability scores.

In order to take the advantage of ensemble process, in this work, we have combined the results obtained from GRNN and PNN models to solve classification problem. The classifiers can then be combined using one of several different level such as abstract level, rank level, and measurement level. In this work, we have used simple ensemble using AND operation which may always lead to an improved performance. We intentionally used AND operation as we want the system to be more stringent in making decision.

## Experimentation and Comparative Analysis

### Dataset

Experiments are performed on a publicly available dataset, created by Joseph Cohen, containing COVID-19 X-ray images [23]. The dataset is composed of training and testing. Furthermore, it is divided into four categories namely COVID-19, normal, pneumonia bacterial, and pneumonia virus. A total of 306 images with a break-down of 69, 79, 79, and 79, respectively for each of the four classes are considered.

### Experimental Setup

The proposed model was implemented in Matlab with Intel Core i7 – 6700 @3.4 GHz and 8GB RAM. We have conducted three types of experiments. First we have considered 2 classes (COVID-19 and Normal), in second type of experiment, we considered 3 classes (COVID-19, normal and pneumonia bacteria), and lastly, we considered all 4 classes (COVID-19, normal, pneumonia bacteria, and pneumonia virus).

**Fig. 2:**
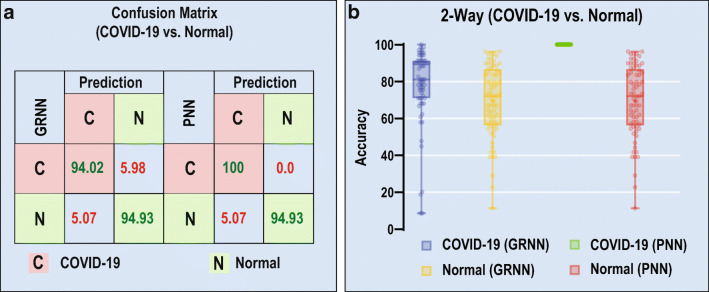
(a) Confusion matrix for 2 class (COVID-19 and Normal). (b) Detection accuracy of individual samples of GRNN and PNN under COVID-19 and Normal cases

To start with, we have randomly selected one image in each category for training purpose, keeping rest of the images for testing. Figure 2(a) shows the confusion matrix for 2 classes using GRNN and PNN approach for randomly selected images. Clear comparison between GRNN and PNN can be noticed when only one-shot (one image) training is used. It is very much necessary to test the significance of each sample in the dataset and to know its contribution for success.

In this regard, each sample in the database is considered for training and remaining samples are used for testing. Figure 2(b) shows the detection accuracy of the proposed GRNN and PNN approaches for both the cases, i.e., COVID-19 and Normal. This helps in understanding the contribution of each sample for the success towards designing a better model. Interestingly, under PNN approach for COVID-19 class, all the samples achieved 100% when trained. Hence, high performing samples are clustered for our next process of classification task.

**Fig. 3:**
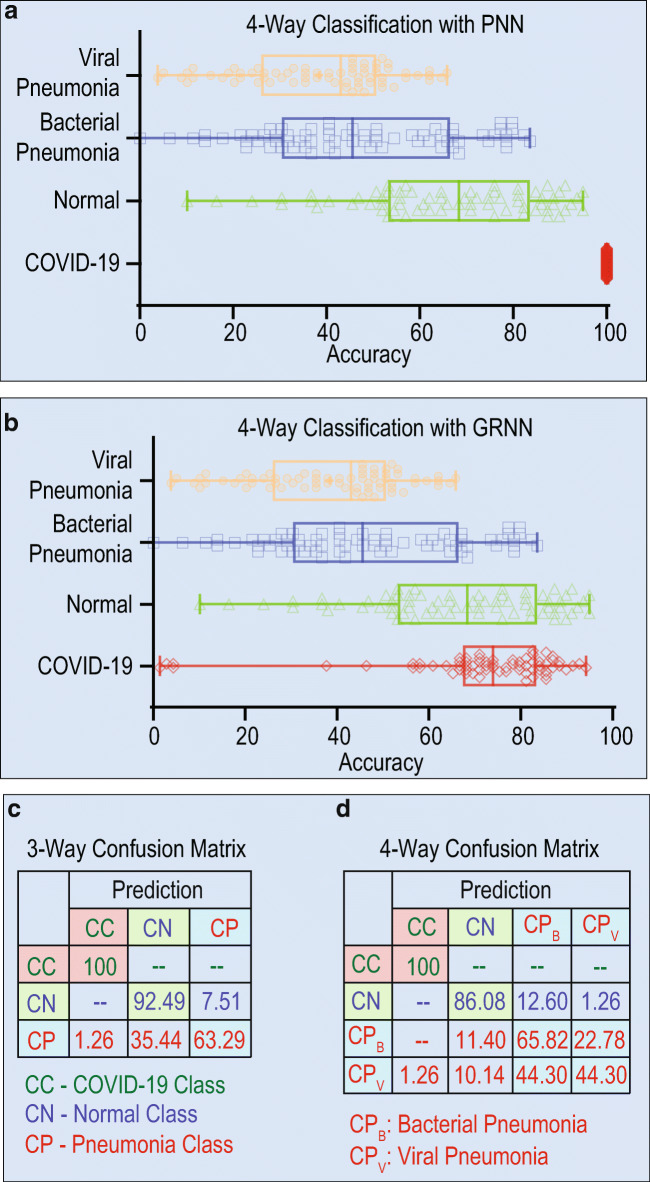
Detection accuracy of individual samples under 4 classes (COVID-19, Normal, Pneumonia Bacteria, and Pneumonia Virus) with PNN (a) and GRNN (b). The Confusion matrices of 3-way and 4-way classifications are shown in (c) and (d), respectively

The second type of experiment is working with all the classes. As the class size increases analyzing the performances of the models are crucial and we are much interested to see the performance behavior. We have conducted the experiment in the similar manner as explained in two classes. Figure 3(a) and 3(b) show comparative results between GRNN and PNN for all four classes (COVID-19, Normal, Pneumonia Bacteria, and Pneumonia Virus). It is very interesting to see how each sample in the database contributes for the detection accuracy.

### Cluster and Ensemble Approach

This section highlights very important aspect of this work, where we cluster samples that performed well in our training phase. From Fig. 3, it is quite evident that the results obtained from the GRNN and PNN have bit of variations. Considering these variations, we have selected the best performing samples for our next level of detection process. Detection process is carried out using ensemble AND operation. The choice of AND operation is to make our decision very stringent and hence we expect both the classifiers to classify to correct category. Table 1 shows the detection accuracy for the samples considered for all four classes using ensemble AND approach. From Table 1, it is evident that the proposed model with only 4 samples (two from each class) for 2 classes was able to achieve 100% detection rate and we wanted our model to correctly distinguish COVID-19 being truly labeled as COVID-19.

**Table 1:** Performance accuracy of the proposed and existing deep models for 2, 3, and 4 classes. The italic entries denote the best results obtained.

Methods	Class	COVID-19	Normal	Pneu. Bac.	Pneu. Vir	Average	Train	Test
Alexnet [31, 32]	2	*100*	*100*	–	–	*100*	130	18
	3	*100*	77.7	77.8	–	85.1	200	27
	4	*100*	64.3	44.4	50	64.67	270	36
Googlenet [31, 34]	2	*100*	*100*	–	–	100	130	18
	3	81.8	75	87.5	–	81.4	200	27
	4	*100*	*100*	70	66.7	84.0	270	36
Resnet18 [31, 33]	2	*100*	*100*	–	–	100	130	18
	3	100	100	64.3	–	81.4	200	27
	4	100	100	50	40	72.5	270	36
Proposed method	2	*100*	*100*	–	–	*100*	*04*	*148*
	Cluster 1 (5 samples)							
	3	100	64.55	68.35	–	77.61	5	227
	Cluster 2 (7 samples)							
	3	100	70.99	68.35	–	79.76	7	227
	Cluster 3 (13 samples)							
	3	*100*	*92.49*	*63.29*	–	*85.23*	*13*	*227*
	Cluster 1 (4 samples)							
	4	94.2	54.43	54.43	44.3	61.84	4	306
	Cluster 2 (5 samples)							
	4	100	54.43	54.43	44.3	63.29	5	306
	Cluster 3 (6 samples)							
	4	100	58.23	53.16	43.04	63.60	6	306
	Cluster 4 (6 samples)							
	4	100	69.62	51.90	41.77	65.80	6	306
	Cluster 5 (29 samples)							
	4	*100*	*86.08*	*65.82*	*44.30*	*74.05*	*29*	*306*

Table 1 also shows the performance for 3 classes. Initially we tried with training five samples and tested our approach and an average of 77.61% accuracy was achieved. Our experiment was continued adding a sample to each class 2 and 3 and an average of 79.76% detection accuracy was reported. Our interest is to improve the accuracy of class 2, i.e., Normal case. In this connection, we added few more samples from classes 1 to 2 and a total of 13 samples were considered in cluster 3. The proposed method outperformed well-known existing approaches and achieved an average of 85.23% and retaining 100% under COVID-19 case.

As the class size increases, it is always interesting to see the effect of the proposed approach. Thus, we also tested our approach on all 4 classes of the images. We started with one sample from each class and a total of 4 samples were considered for training. An average detection rate of 61.84% was achieved and the detection rate for pneumonia virus is very less compared to others. As mentioned, we always wanted our model to correctly distinguish COVID-19 being truly labeled as COVID-19. Hence, in this connection, we added one sample from class 1 and tested our approach with five samples. An average of 63.29% and achieved 100% under class 1. We continued our clustering approach by adding few more samples to the existing database. From Table 1, it is quite evident that detection accuracy is improved by 10% under normal case with an average detection rate of 65.80%. There is a high degree of similarity between pneumonia bacteria and pneumonia virus; thus, correctly classifying them is a real challenge. We continued our approach with added few more samples to all the classes and an average of 74.05% with substantial improvement under normal case is resorted. There is always a scope to further enhance the results with different cluster sizes and in this work, we have limited our experiment to cluster 5. Certainly, the proposed idea of one shot with cluster approach has a considerable impact in detection process. Figure 3(c) and 3(d) show the confusion matrices for 3 and 4 classes, respectively.

### Comparative Study

Recently, researchers are now focusing on developing some AI models to overcome the issues and have revealed some significant discoveries in imaging studies of COVID-19. In this work also, we compared our results with that of well-known deep learning models such as AlexNet [24], GoogleNet [25], and ResNet [26]. The compared CNN models (refer to Table 1) in this work had a few numbers of layers compared to large CNN models such as Xception, Densenet, and Inceptionresnet which consist of 71, 201, and 164 layers accordingly. The choice of these models will reflect on reducing the training time and the complexity of the calculation. From the results, it is quite evident that the proposed model outperformed all other deep models. The compared deep models use 80% of the dataset for training and only 20% for testing purpose. We also compared our approach with well-known deep convolution neural networks, i.e., COVID-Net [7]. In COVID-Net [7], a total 13,975 chest X-ray images are considered for experimentation purpose, out of which 100 images are used for testing. The accuracy reported is 93.3% under 3 classes. Our proposed method has performed reasonably well for 2 classes and is being very competitive under 3 classes with only 13 samples trained and 227 images tested. This work is an initial effort in understanding the behavior of the existing neural network models with a small dataset. There is still a long way to understand and analyze the models for different applications and also with huge data and class size.

## Discussion and Conclusion

This paper proposes one-shot cluster-based approach for efficient classification of COVID-19 chest X-ray images. The main objective was to study the ability of learning image categories at a fast pace and with less sample size. Certainly, the proposed idea has considerable impact when compared to existing deep learning models. Deep learning models are computationally expensive and require huge data for training. Conventional networks such as GRNN/PNN have generalization and convergence characteristics. For this purpose, we tested our model with COVID-19 chest X-ray images. The proposed cluster-based one-shot learning is shown to be more effective on GRNN and PNN ensembled model to distinguish COVID-19-affected images from that of the other three classes. It has also been experimentally observed that the model has a superior performance over contemporary deep learning architectures. The concept of one-shot cluster-based learning is being first of its kind in literature, expected to open up several new dimensions in the field of machine learning which require further researching for various applications.
